# Smoking Cessation Treatment among Rural Individuals

**DOI:** 10.47496/nl.CDM.2020.01.01

**Published:** 2020-11-11

**Authors:** Steven S. Coughlin

**Affiliations:** 1 Department of Population Health Sciences, Medical College of Georgia, Augusta University, Augusta, Georgia, USA; 2 Institute of Public and Preventive Health, Augusta University, Augusta, Georgia, USA

**Keywords:** randomized controlled trials, rural, smoking cessation

## Abstract

Rural population in the U.S. have higher smoking prevalence rates and consume a higher number of cigarettes per day. Socioeconomically disadvantaged smokers, such as those who reside in rural areas, are less likely to use and have access to evidence-based tobacco cessation treatments than the general population of smokers. Randomized controlled studies are needed to examine the effectiveness of evidence-based smoking cessation interventions among rural residents. Of particular interest are interventions that overcome barriers to smoking cessation treatment such as poor access to primary care, travel, time, lack of health insurance, an inability to pay out-of-pocket expenses for pharmacotherapy, and communal norms that influence smoking cessation.

Tobacco use remains the leading cause of preventable mortality and morbidity in the U.S. and is responsible for nearly 443,000 deaths annually [1, 2]. Rural populations in the United States have higher smoking prevalence rates than their urban counterparts and consume a higher number of cigarettes per day [3–5]. In the last 10 years, the proportion of smokers in rural populations has remained stable or even increased, emphasizing the need for further efforts to assess smoking cessation treatment access in rural communities [3, 6]. Ramsey *et al.* [3] noted that a significant proportion of health disparities in rural populations could be eliminated with low-barrier, easy access treatment delivery methods for smoking cessation. Tobacco cessation interventions for rural residents have been minimally tested in randomized controlled trials [7].

Socioeconomically disadvantaged smokers, such as those who reside in rural areas, are less likely to use and have access to evidence-based tobacco cessation treatments (pharmacotherapy and behavioral skills counseling) than the general population of smokers [2, 8]. Other barriers include a lower likelihood of receiving preventive care services, infrequent physician visits, difficulty taking time from work for cessation services, travel time, lack of health insurance, an inability to pay out-of-pocket expenses for pharmacotherapy, limited local smoking cessation programs, lack of knowledge of existing resources for smoking cessation, and communal norms that influence smoking cessation [9, 10].

Relatively few studies have examined the feasibility, acceptability, or effectiveness of smoking cessation treatment among rural residents. Ramsey *et al.* [3] examined the effect of an electronic health record-based smoking cessation module on patient receipt of evidence-based cessation care. They retrospectively studied 479,798 patients across 766 clinics in the greater St. Louis, southern illinois, and mid-Missouri regions. Smoking prevalence was higher in rural versus urban clinics (20.7% vs. 13.9%, p < 0.0001). Rural smokers were nearly three times less likely than their urban counterparts to receive any smoking cessation treatment (9.6% vs. 25.8%, p < 0.0001). Schorling *et al.* [11] conducted an 18-month trial of church-based smoking cessation interventions for rural African Americans who were residents of two Virginia counties. Up to two smoking cessation counselors were trained from participating churches. At follow-up, the smoking cessation rate in the intervention county was 9.6% and in the control county 5.4% (P = 0.18). Schoenberg *et al.* [12] conducted a faith-based smoking cessation program in rural Appalachia, involving 590 smokers in 26 rural churches randomized to early and delayed intervention group. in qualitative evaluation, participants overwhelmingly provided positive assessments of the program. Stewart *et al.* [13] conducted a quasi-experimental study of a tobacco cessation program in rural, medically underserved, blue collar employees. A statistically significant difference was seen over time between the intervention and control worksites on knowledge of the dangers of tobacco (P < 0.0001). A substantial body of evidence indicates that quit lines for cessation are effective in helping people to people to stop smoking. However, there is a paucity of research on quit lines’ effectiveness in rural populations. Vander Weg *et al.* [14] conducted a pilot randomized controlled trial of a smoking cessation intervention for rural Veterans. Participants were randomly assigned to an individually-tailored telephone intervention or to treatment provided through their state tobacco quit-line. Twelve week quit rates did not differ significantly by group (tailed = 39%, quit-line referral = 25%, odds ratio [OR] = 1.90, 95% confidence interval [CI] 0.56, 5.57). Six-month quit rates for the tailored and quit-line referral groups were 29% and 28%, respectively (OR = 1.05, 95% CI 0.35, 3.12).

Most cigarette smokers want to quit smoking, and about 50% make a quit attempt each year, but only 6% achieve long-term cessation [1]. Effective treatments exist, and the evidence-based clinical practice guideline recommends a combination of behavioral skills counseling and pharmacotherapy because this multi-modality approach is most effective for helping smokers quit over the long-term [15]. The U.S. Public Health Service Clinical Practice Guideline for the Treatment of Tobacco Dependence provides best practice standards for treating tobacco dependence [15]. Techniques stemming from behaviorally based counseling models, including motivational enhancement and skills training, are effective for tobacco cessation [16]. The provision of social support is also beneficial. Pharmacotherapies for tobacco cessation include nicotine replacement therapy (nicotine patch, gum, inhaler, spray, and lozenge), the antidepressant bupropion, and the newest agent Varenicline (Chantix).

Randomized controlled studies are needed to examine the effectiveness of evidence-based smoking cessation interventions among rural residents. Of particular interest are interventions that overcome barriers to smoking cessation treatment such as poor access to primary health care, travel time, lack of health insurance, an inability to pay out-of-pocket expenses for pharmacotherapy, and communal norms that influence smoking cessation.
